# Machine learning prediction of post-CABG atrial fibrillation using clinical and pharmacogenomic biomarkers

**DOI:** 10.3389/fmed.2025.1650700

**Published:** 2025-09-11

**Authors:** Lei Hua, Jingxian Han, Siqi Zhang, Zhiying Li, Hui Qiao, Bin Yang, Xiangguang Meng

**Affiliations:** ^1^Henan Key Laboratory of Cardiac Remodeling and Transplantation, The 7th People’s Hospital of Zhengzhou, Zhengzhou, Henan, P.R. China; ^2^Department of Medical Laboratory, The 7th People’s Hospital of Zhengzhou, Zhengzhou, Henan, P.R. China; ^3^Department of Pharmacy, The 7th People’s Hospital of Zhengzhou, Zhengzhou, Henan, P.R. China

**Keywords:** coronary artery bypass grafting, postoperative atrial fibrillation, machine learning, prediction model, gaussian naive bayes

## Abstract

**Background:**

Postoperative atrial fibrillation (POAF) is a frequent complication following coronary artery bypass grafting (CABG), significantly impacting patient prognosis and healthcare costs. This study aimed to develop an integrated predictive model for POAF risk stratification to optimize clinical management.

**Methods:**

We retrospectively analyzed 2,528 patients undergoing 21-gene pharmacogenetic testing for cardiovascular therapy. After stringent data curation, 576 CABG patients were enrolled and randomly allocated into training and test sets. Eight machine learning algorithms were trained using clinical variables and genetic variants. An independent validation set was performed on 61 patients from a subsequent 1,075-patient cohort of 21-gene pharmacogenetic testing.

**Results:**

Eight machine learning algorithms were trained, tested, and validated, with the Gaussian Naive Bayes (GNB) model demonstrating robust performance (Accuracy: 0.81 in test set and 0.79 in independent validation set). SHapley Additive exPlanations analysis identified four key predictors: multivessel CABG (CABGVx ≥ 3), history of heart failure (HFHx), rs5219 (*KCNJ11*), and prolonged bypass duration (CABGTime). To facilitate clinical translation, we developed an accessible web-based tool (https://www.xingyeyard.site/cabg/) for real-time POAF risk stratification.

**Conclusion:**

This GNB-based classifier synergistically integrates Pharmacogenomic and clinical predictors to predict POAF risk following CABG. The combination of rigorous validation and user-centered design positions this model as a valuable clinical decision-support tool for optimizing personalized perioperative care.

## Graphical Abstract

GNB, gaussian naive bayes; KNN, K-Nearest Neighbors; LR, logistic regression; MLP, multilayer perceptron; RF, random forest; SVM, support vector machine; TabTF, TabTransformer; XGB, extreme gradient boosting. 
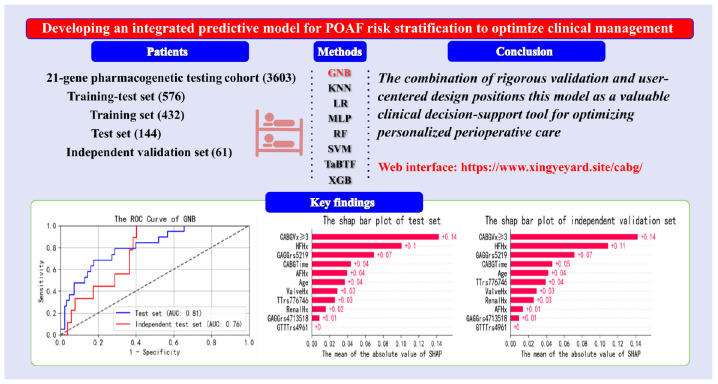

## Background

Postoperative atrial fibrillation (POAF) is a common complication following heart surgery, with an incidence of approximately 20% after coronary artery bypass grafting (CABG) (1). It typically occurs 2–4 days postoperatively, is often asymptomatic, and may resolve spontaneously. However, despite this, the complication still has a significant impact on the patient’s quality of life and can even be life-threatening. It also increases hospital stay and medical costs. Currently, the mechanisms underlying POAF are controversial, and effective preventive strategies are lacking. Therefore, predicting and treating POAF has become a hot topic in cardiovascular research.

With the advancement of pharmacogenomics research, there is a growing recognition of the importance of genetic factors in cardiovascular diseases and their pharmacological treatment. Numerous studies have shown that polymorphisms in specific genes not only affect the efficacy of cardiovascular drugs but may also be closely associated with the occurrence of POAF after CABG. For example, genetic polymorphisms in platelet membrane glycoprotein IIb/IIIa complex, cytochrome P450 (CYP) enzyme system, and others may influence the efficacy of antiplatelet drugs such as aspirin and clopidogrel, thereby affecting the risk of POAF after CABG (2). Xue et al. suggested that polymorphisms in the apolipoprotein E (*ApoE*) gene are associated with POAF and cardiac injury following CABG (3).

Additionally, eight machine learning (ML) models related to the prediction of POAF have been developed. Tan et al. combined gene expression information from 139 CABG patients and used LASSO regression to build a POAF prediction model (4). Although the sample size was relatively small, the study suggested that combining genetic and clinical information could be a promising approach for POAF prediction. ML has been applied across various medical fields, particularly in cardiovascular medicine, for tasks such as diagnosis, treatment optimization, and prognosis prediction. The research data encompasses diverse medical information, extending beyond textual data (5). The algorithms used often outperform traditional statistical methods. Some studies suggest that ML is more effective than standalone clinical or imaging methods in predicting cardiovascular mortality or all-cause mortality (6).

In summary, regarding the prediction of the occurrence of POAF after CABG, most of the literature we reviewed had small sample sizes and single models. In particular, studies either considered only clinical factors or only genetic factors, and we have not come across any research that integrates both analyses. Therefore, in this study, we utilized eight ML-based algorithms and incorporated a relatively large sample size to combine cardiovascular gene variant loci with clinical data in order to construct a prediction model for POAF after CABG. Additionally, we developed a corresponding online prediction software to provide insights for potential complications in these patients and facilitate early decision-making interventions.

## Materials and methods

### Study subjects

The study cohort comprised 576 patients who underwent 21-gene cardiovascular pharmacogenomic testing at Zhengzhou Seventh People’s Hospital (07/2022-04/2024). Inclusion criteria: East Asian adults receiving first-time CABG. Exclusion criteria: age <18 or >100 years, non-CABG cases, repeat CABG, cancer, or cardiac structural abnormalities. These formed the training-test set for ML development. An independent validation set included 61 CABG patients (05-12/2024).

### POAF diagnosis criteria

Post-cardiac surgery and before discharge, atrial fibrillation (AF) or atrial flutter (AFL) changes are identified in one or more electrocardiogram (ECG) leads, lasting at least 30 s. If the ECG recording duration is less than 30 s but shows continuous AF or AFL changes throughout the entire recording period, POAF can still be diagnosed.

### Clinical data collection

The following patient data were collected: Sex, Age, Height, Weight, Blood type, Smoking, Drinking, Diabetes, Hypertension, history of kidney disease (RenalHx), history of liver disease (LiverHx), history of lung disease (LungHx), history of stroke (StrokeHx), history of valvular disease (ValveHx), history of myocardial infarction (MIHx), history of angina pectoris (APHx), history of coronary artery disease (CHDHx), history of heart failure (HFHx), history of AF (AFHx), history of thyroid disease (ThyroidHx), history of percutaneous coronary intervention (PCIHx), medication history (AnticoagulantHx, LipidMedHx, AntiDiabeticHx, and BPMedHx), multivessel CABG (CABGVx), and prolonged bypass duration (CABGTime).

### Cardiovascular and cerebrovascular personalized medication 21-gene testing

A 5 mL venous blood sample was collected from each patient and subjected to multiple PCR reactions. For detailed operational steps, please refer to Supplementary Method 1. Afterward, gene genotyping data was obtained using a time-of-flight mass spectrometer. The pharmacogenomic profiling encompassed twenty-one clinically actionable loci. More detailed information about these genes and variant sites is provided in Supplementary Table 1.

### Model construction and evaluation

After data cleaning, eight ML models were constructed, including Tree Models: Random Forest (RF) and XGBoost (XGB), Deep Learning Models: Multilayer Perceptron (MLP) and TabTransformer (TabTF), Frequency Model: Gaussian Naive Bayes (GNB), Regression Model: Logistic Regression (LR), Kernel Model: Support Vector Machine (SVM), and Distance Voting Model: K-Nearest Neighbors (KNN). For more details, please refer to Data Cleaning, Clinical Data Cleaning and Feature Dimensionality Reduction, Variant Data Cleaning and Feature Dimensionality Reduction, and Model Construction and Evaluation in Supplementary Method 2.

In this study, the training-test set was randomly split into training and test sets at a ratio of 0.25. Given that the target variable exhibits class imbalance, we performed oversampling of the minority class in the training set using the SMOTE algorithm. For non-tree models, we use the StandardScaler function to standardize the feature variable values. All model construction and hyperparameter optimization were performed using the GridSearchCV tuning function and a custom module, with 10-fold cross-validation. For the GridSearchCV function from the sklearn package, the evaluation metric parameter (scoring) was set to “accuracy”.

Notably, to enhance the representation capability for structured tabular data, this study incorporated the relatively recent TabTF model. The hyperparameter optimization process was customized into a dedicated code segment (detailed code is provided in Supplementary Code 1), with the accuracy metric of 10-fold cross-validation designated as the optimization objective. Considering hardware constraints, we implemented performance optimization via the randomized parameter sampler (ParameterSampler), setting the primary iteration count parameter to 30. In total, the critical optimization parameters comprised 10 variables spanning 256 combinatorial configurations, including: Embedding dimensions (8, 16), Attention head counts (2, 4), Transformer encoder depths (1, 2), Feedforward network hidden dimensions (64, 128), Learning rates (0.001, 1e-4), Batch sizes (16, 32), Dropout rates (0.1, 0.2), Weight decay coefficients (1e-4, 1e-5), Training epochs (50), Random seed (377), with an early stopping patience parameter set to 5.

The final generalization ability was verified using an independent validation set. The optimal model selection was primarily based on Accuracy and the area under the ROC curve (AUC) of the test set.

The explanation method for the optimal model was based on the SHAP package. The best feature contributions of the model were determined using waterfall plots, force plots, decision plots, bar plots, and heatmaps following SHAP values. Based on the overall framework outlined above, we developed a program that can predict the risk of POAF occurrence in patients undergoing CABG surgery, which is accessible online via PC or mobile devices.

### Statistical

Statistical analysis was performed using R. Non-normally distributed continuous variables were reported as median (Q1-Q3) and compared using Mann-Whitney U test. Categorical variables were presented as counts and analyzed by χ^2^ or Fisher’s exact test. Clinical variables with *P* < 0.05 were considered significant. For genetic variant screening in predictive modeling, we used *P* < 0.10 threshold to include more polymorphic loci while maintaining model performance (7, 8), as individual feature-target associations showed limited impact on overall predictive accuracy.

## Results

### General characteristics of patients

**FIGURE 1:**
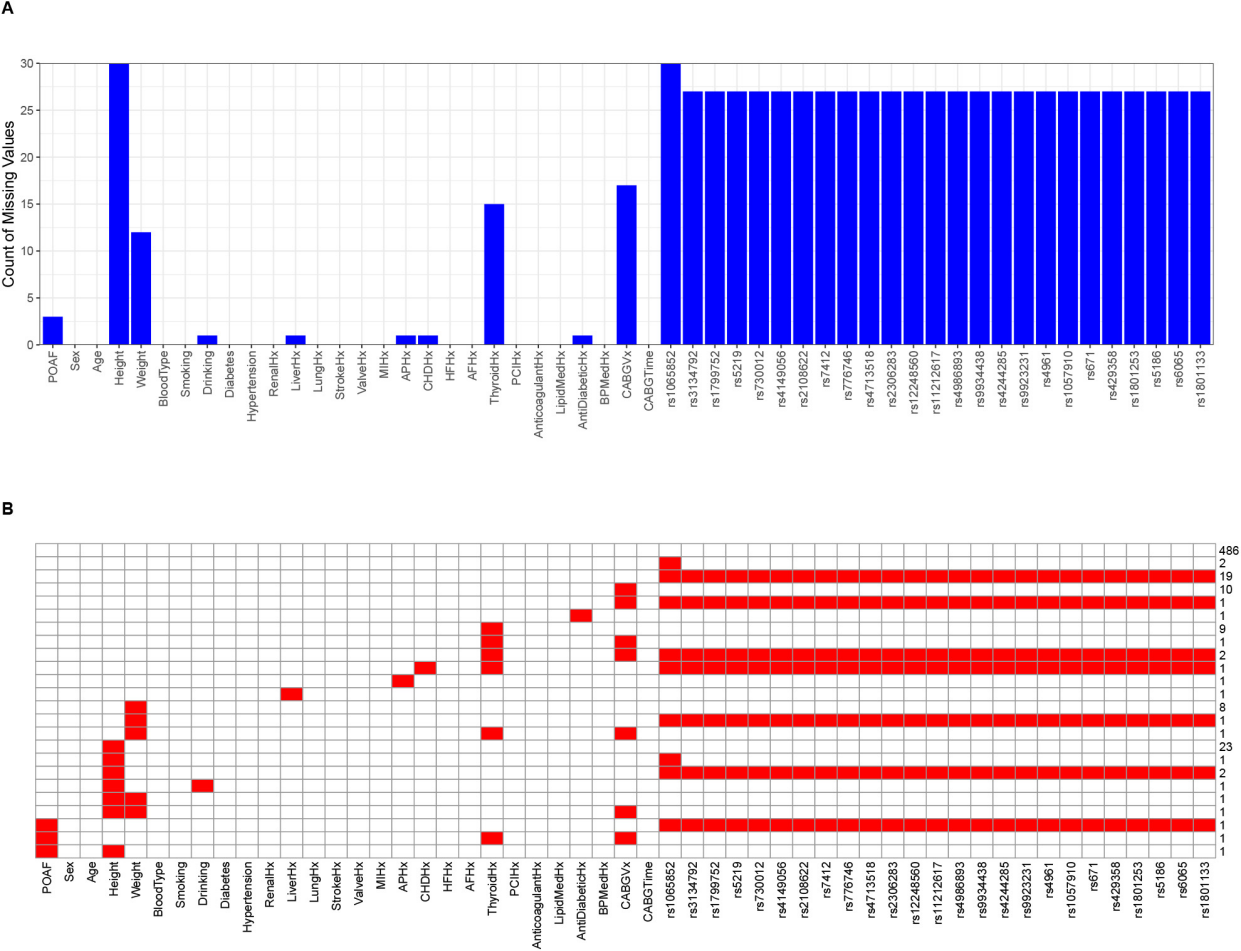
The final distribution of missing data before imputation of 53 feature variables for 576 patients. **(A)** The bar chart displaying the number of missing values. The horizontal axis represents feature variables, and the vertical axis represents the number of missing values. **(B)** The heatmap displaying the missing values. Red cells represent the amount of missing data, corresponding to the scale values on the right vertical axis, while the horizontal axis represents feature variables.

The final dataset comprised 576 CABG patients (478 SR, 98 AF). Pre-imputation missing data (max = 30) are shown in Figures 1A, B. After cleaning, we retained 26 clinical variables (Supplementary Table 2) and 25 genetic variants (Supplementary Tables 3, 4), excluding three biased SNPs (rs10306114, rs1799853, rs5918). HWE testing (*P* > 0.05) was performed only on non-biased variants. The pre-imputation genotype frequency data for all variants are provided in Supplementary Table 5.

After feature reduction, seven clinical characteristic variables were retained, including Age, RenalHx, ValveHx, HFHx, AFHx, CABGVx, and CABGTime, all of which showed statistical differences (*P* < 0.05). Four genetic variants (rs4961, rs5219, rs776746, and rs4713518) showed statistically significant associations (*P* < 0.10) and exhibited minor allele frequencies ranging from 0.29 to 0.50, meeting the threshold for common polymorphisms in the study population. In addition, since the number of variables screened by the χ^2^ or non-parametric tests was exactly suitable for constructing learning models, we did not proceed with further screening using Lasso regression.

**FIGURE 2:**
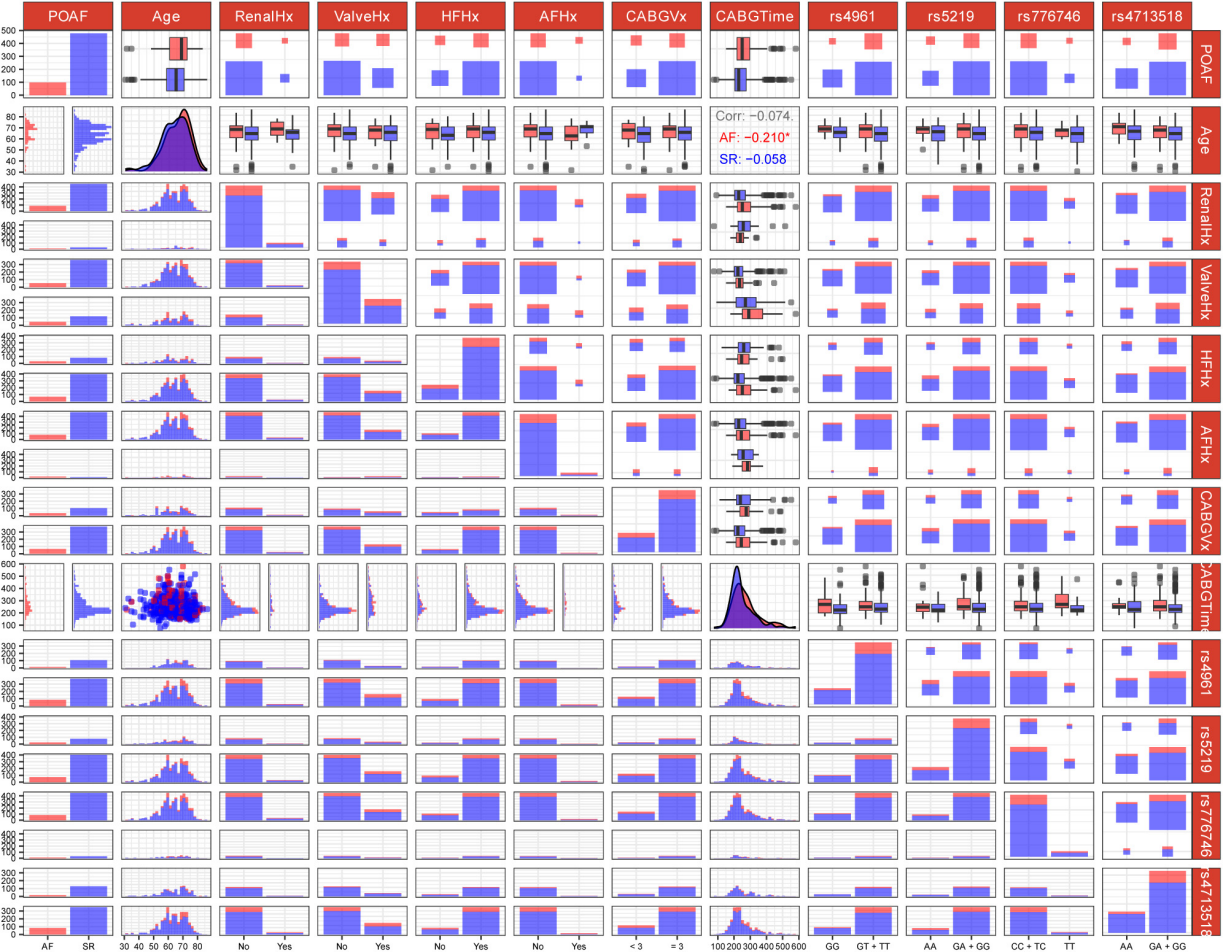
Genotype-phenotype association matrix of genetic variants and clinical factors related to post-operative atrial fibrillation (POAF). The matrix displays pairwise associations between features. Each row and column represent a variable, with diagonal cells showing distribution patterns. Off-diagonal cells visualize relationships between variable pairs using distribution plots, box plots, scatter plots, and heat maps. Blue and red color coding distinguishes between case/control groups or “Yes/No” status. For instance, in matrix cell (1, 1), red indicates POAF patients with “Yes” status while blue represents “No” status POAF patients. Clinical variables include age and medical history factors (RenalHx, renal disease history; ValveHx, valve disease history; HFHx, heart failure history; AFHx, atrial fibrillation history; CABGVx, coronary artery bypass graft history; CABGTime, time-related CABG parameters). Genetic markers analyzed include rs4961, rs5219, rs776746, and rs4713518. Some cells annotate the Pearson correlation coefficients between two continuous variables. For instance, the three lines of text in matrix cell (2, 8) represent the correlation coefficients of Age with POAF across three scenarios: AF/positive cases, SR/negative cases, and AF + SR cases. Asterisks (*) denote statistically significant correlations.

Consequently, these 11 features were selected as predictive variables for ML model development. Their distributions are visualized in Figure 2, with Age and CABGTime being the sole continuous variables. As demonstrated in cell (2, 8) of the figure, Age exhibited negligible linear correlations with POAF across all cohort groups, as evidenced by Pearson’s r values of −0.074 in the AF cohort (positive cases), −0.210 in the SR cohort (negative cases), and −0.058 in the combined group (AF + SR).

We assessed multicollinearity between continuous variables (Age and CABGTime) using VIF analysis, finding VIF = 1.00 for both (VIF < 5 indicates acceptable collinearity). Categorical variables weren’t evaluated due to: (1) satisfactory model performance suggesting minimal collinearity impact; (2) VIF’s linear relationship assumption; and (3) ML algorithms’ inherent handling of categorical variable interactions.

### Prediction model construction

The dataset used for model learning consisted of 478 SR and 98 AF patients, respectively, indicating a biased target variable. After SMOTE oversampling, the training set contained 353 SR and 353 AF cases. The eight models were built using the balanced training set. The hyperparameter configurations for each model are provided in Supplementary Table 6, and their optimal parameter combinations are listed in Supplementary Table 7.

### Evaluation of prediction models

**FIGURE 3:**
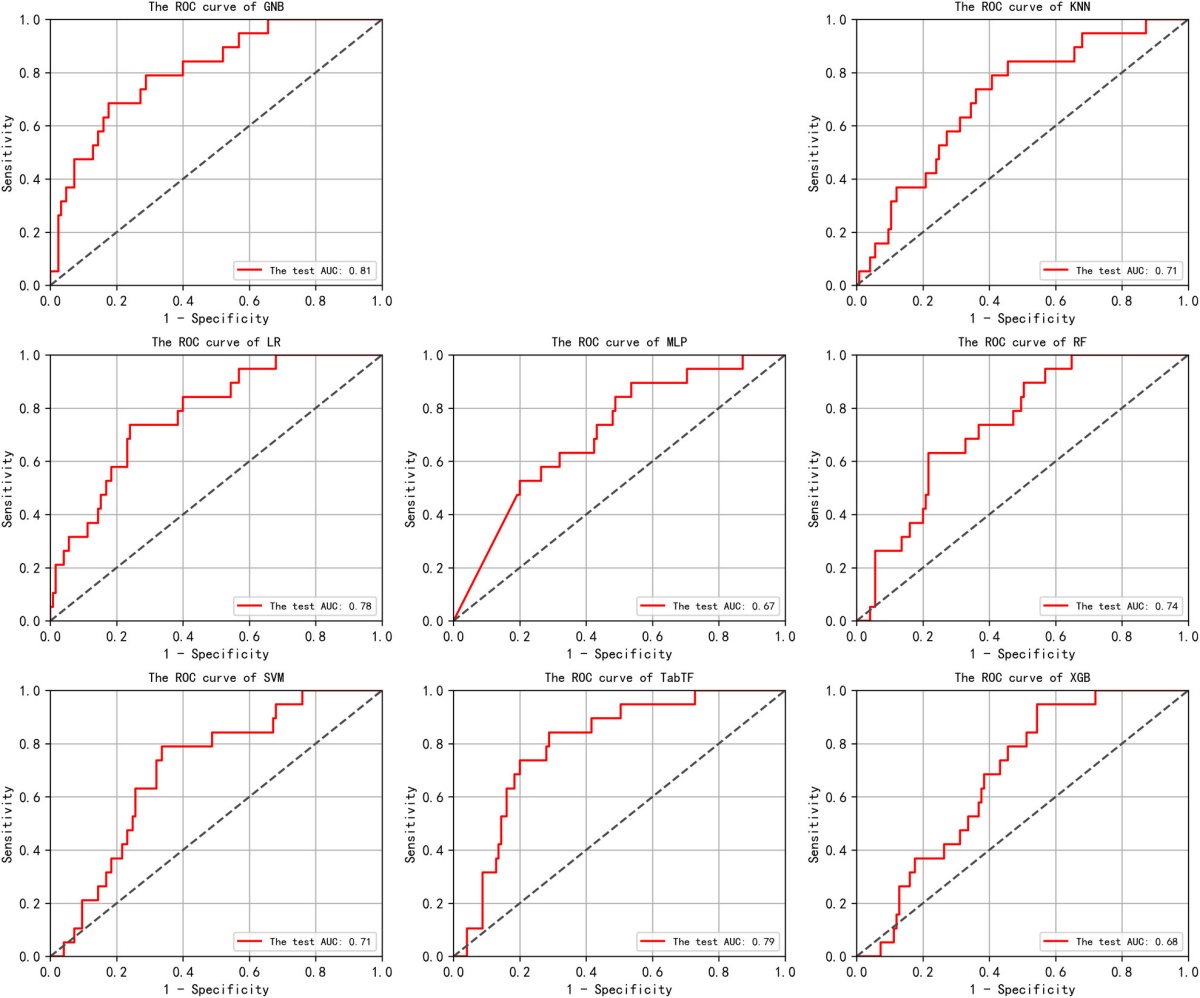
In the test set, the Receiver Operating Characteristic (ROC) curves for the results of eight machine learning algorithms are shown. The horizontal axis plots the False Positive Rate (FPR), while the vertical axis plots the True Positive Rate (TPR). An Area Under the Curve (AUC) of 0.5 indicates random classification performance, while values close to 1 indicate higher accuracy. An ROC curve that approaches the top-left corner suggests that the model achieves a good balance between high true positive rate and low false positive rate, indicating effective discrimination between positive and negative classes.

Supplementary Table 8 presents the accuracy and AUC values for all eight models on the test set, along with the optimal performance metrics on the independent validation set. Among these, the GNB model exhibited the strongest predictive performance, achieving an accuracy of 0.81 and an AUC of 0.81 (95% CI: 0.70–0.91) in the test set. While the recently emerged deep learning algorithm TabTF demonstrated improved performance over MLP with an accuracy of 0.76 and an AUC of 0.79 (95% CI: 0.69–0.88), it nonetheless remained inferior to GNB. In contrast, XGB–frequently recognized for its remarkable performance in Kaggle competitions–achieved only 0.71 accuracy and an AUC of 0.68 (95% CI: 0.54–0.77). This performance gap may be attributable to the limited sample size. Consequently, it was selected as the optimal model for further validation on the independent validation set and for SHAP-based feature interpretation. The GNB model demonstrated robust generalization capability in the independent validation set, maintaining an accuracy of 0.79 and an AUC of 0.76 (95% CI: 0.62–0.89). The receiver operating characteristic (ROC) curves for all eight models are illustrated in Figure 3, providing a comparative assessment of their binary classification performance.

### Interpretation of the optimal prediction model features

**FIGURE 4:**
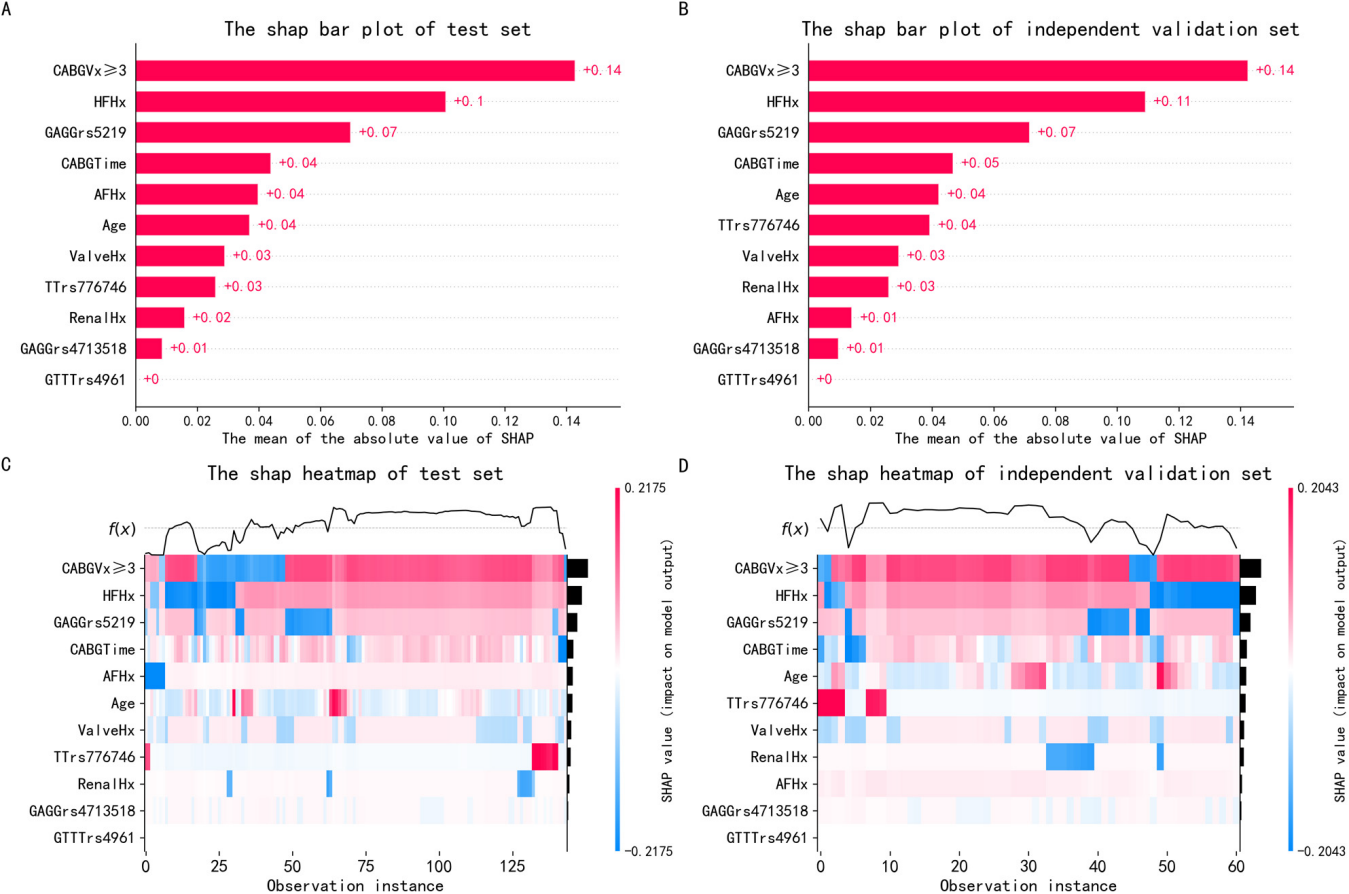
SHAP visualizations for the optimal GNB model. **(A,B)** Bar plots showing mean absolute SHAP values (x-axis) for test (*n* = 144) and independent validation (*n* = 61) sets, where taller bars indicate greater feature importance. **(C,D)** Heatmaps displaying individual predictions (top curves) and feature impacts (bottom vertical axis) per sample (horizontal axis). Color intensity reflects effect magnitude (blue = positive, red = negative), with the gradient bar quantifying SHAP values.

Our SHAP analysis revealed strong consistency in feature importance rankings between the test set and independent validation set. The SHAP bar plot (Figures 4A, B) demonstrated that the top four predictive features maintained stable contribution weights: CABGVx ≥ 3 (Test: 0.14, Independent: 0.14), HFHx (Test: 0.1, Independent: 0.11), GAGGrs5219 (Test: 0.07, Independent: 0.07), and CABGTime (Test: 0.04, Independent: 0.05). Complementing these findings, the SHAP heatmap (Figures 4C, D) visualized individual patient-level explanations, where: Color intensity represents the magnitude of feature impact, Hue indicates directional effect (positive/negative prediction influence), the same four features consistently showed the strongest predictive weights.

The remarkable agreement between the test and independent validation sets in bar plot and heatmap interpretation methods strongly supports the model’s robust generalization capability. This consistency across different explanation visualizations and datasets enhances our confidence in the model’s clinical applicability.

**FIGURE 5:**
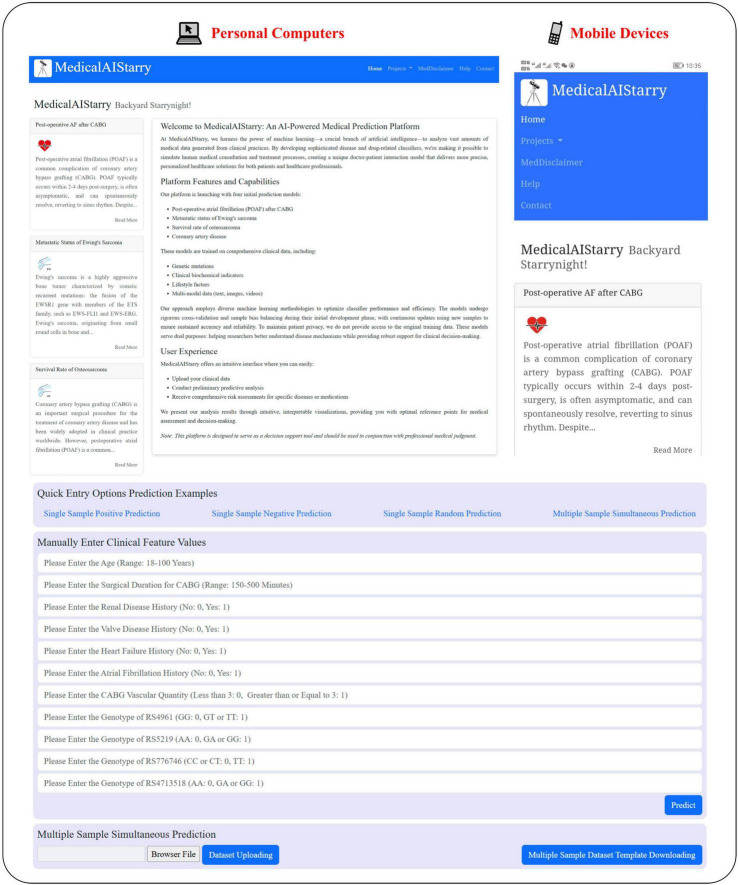
Risk prediction program for POAF occurrence in patients after CABG surgery. Website: https://www.xingyeyard.site/cabg/

In addition to the global interpretability visualizations, our online platform–MedicalAIStarry– provides patient-specific local interpretation diagrams (including waterfall plots, force plots, and decision plots) for each POAF case. It is currently accessible via PC or mobile devices at the website: https://www.xingyeyard.site/cabg/ (Figure 5).

## Discussion

**FIGURE 6:**
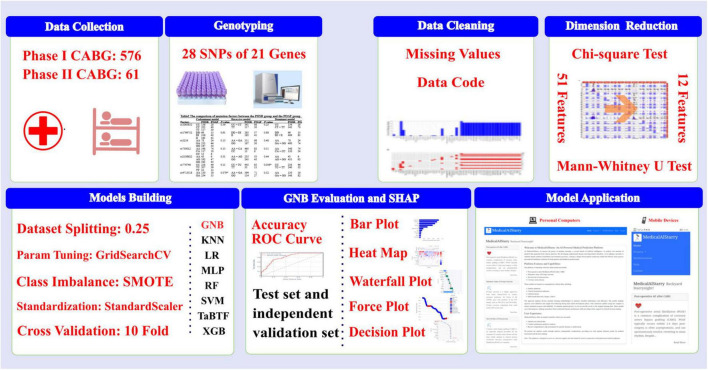
The flowchart of this study. GNB, gaussian naive bayes; KNN, K-Nearest Neighbors; LR, logistic regression; MLP, multilayer perceptron; RF, random forest; SVM, support vector machine; TabTF, TabTransformer; XGB, extreme gradient boosting.

In this study, based on a cohort of CABG patients who underwent routine pharmacogenetic testing upon admission in our center, we utilized the data from existing clinical records and genetics test results to construct eight ML models, in which the optimal GNB robustly predicts the probability of POAF occurrence. The subsequent SHAP analysis results revealed the top four contributing feature variables: CABGVx ≥ 3, HFHx, GAGGrs5219, and CABGTime. Ultimately, an online risk prediction program for POAF after CABG surgery was developed. This program integrates both genetic and clinical features, requiring only 11 input variables to predict the risk of POAF occurrence in individual or multiple CABG patients, providing a personalized risk prediction interface for patients. The detailed research procedure is illustrated in Figure 6.

It has been reported that after the age of 60, there is an increase in elastic and collagen tissues in the conduction system, along with fat accumulation around the sinoatrial node, leading to a significant reduction in the total number of pacemaker cells in the sinoatrial node. This may increase the susceptibility of elderly individuals to POAF (9). Renal dysfunction is a known risk factor for POAF after CABG (10). Rheumatic aortic valve disease and degenerative aortic valve lesions are independent risk factors for POAF development. The prolonged course of these conditions may increase the release of reactive oxygen species, which further induce myocardial electrical remodeling, manifesting as a shortening of the action potential effective refractory period, thereby triggering POAF (11).

In this study, a history of AF and heart failure were also found to be important variables in predicting the development of POAF. Previous studies have reported that AF is associated with pathological abnormalities in the left atrium, including atrial enlargement, fibrosis, impaired calcium handling, electrical remodeling, and decreased function, while heart failure is associated with myocardial damage, excessive cardiac load, arrhythmias, and other factors. Therefore, AF and heart failure can influence each other, leading to worsening of the condition (12). In addition, the occurrence of POAF after CABG is the result of multiple factors. Preoperative cardiac condition, postoperative cardiac function, and certain factors during the surgery may all contribute to the development of POAF (13).

Studies have shown that POAF is associated with the number and duration of CABG (14). In our study, we found that the number of grafts (≥3) ranked first in the feature importance of the GNB model, and the surgery duration ranked fourth. This suggests that these factors involved in the surgical process may indeed be related to the occurrence of POAF and play an important role in predicting it. This may be linked to the following mechanism: the greater the number of vessels involved in the surgery, the higher the complexity of the operation, and the longer the surgery time and trauma, which could increase the risk of POAF.

In recent years, AF has increasingly been recognized as a hereditary disease, and genome-wide association studies have identified several common variants associated with AF. Studies have shown that there are multiple single nucleotide polymorphism (SNP) sites on chromosome 4q25 associated with AF, among which rs2200733 and rs10033464 are two common loci (15). However, previous studies have suggested that predictive variables exhibiting strong univariate associations with the target outcome do not necessarily translate into enhanced predictive performance (8). Furthermore, our center routinely conducts pre-CABG pharmacogenomic profiling for hospitalized patients, which enabled the identification of candidate genetic variants informing this study design.

Based on four genetic models, four loci (rs5219, rs776746, and rs4713518, and rs4961) were incorporated into the ultimately predictive model of GNB. Notably, rs5219 demonstrated the highest interpretative weight in the four variants, whereas rs4713518–despite demonstrating the most significant association (*P* = 0.034)–exhibited the third-highest explanatory weight. This observation corroborates existing literature demonstrating that features lacking statistical significance in univariate analyses may still contribute substantially to predictive models, a phenomenon further validated by recent reports highlighting the discordance between statistical significance and predictive utility (7).

Rs5219 is located in the *KCNJ11* gene, which resides on chromosome 11. This gene contains one exon and encodes the Kir6.2 subunit of the ATP-sensitive potassium channel (KATP). The SNP database from NCBI shows that the variant frequency of rs5219 does not differ significantly across different ethnic groups, with the minor allele frequency being 0.36 in Europeans, 0.37 in Asians. In this study, the variant frequency at this site was 0.42, which slightly deviates from the aforementioned results, suggesting heterogeneity between the disease and healthy populations. Studies have reported that rs5219 is associated with susceptibility to ischemic heart disease, with the G/A genotype being more common in patients with coronary artery disease, while the G/G genotype is more prevalent in patients with coronary microvascular disease (16). Strutynskyi et al. (17) found that rs5219 is one of the risk factors for heart failure (17). Although there is no direct evidence to suggest that the rs5219 polymorphism is the primary pathogenic factor for POAF, we observed that the rs5219 site has a significant predictive weight in the GNB model. The potential mechanism may involve the effect of this polymorphism on cardiac electrical activity or myocardial cell function. Furthermore, Yi et al. found that rs776746 increased susceptibility to ischemic stroke and was associated with arterial thrombotic events in stroke patients (18). Studies have shown that in Taiwanese adults, for individuals carrying the rs4713518 AA genotype, the interaction between hyperlipidemia and gender affects the risk of gout (19). Rs4961 is an SNP located on the α-adducin (*ADD1*) gene. Dysfunction of *ADD1* can lead to hypertension by enhancing sodium reabsorption in renal tubular epithelial cells, while pharmacological inhibition of *ADD1* can significantly lower blood pressure (20). Therefore, the functional *ADD1* polymorphism is considered a potential genetic marker for hypertension.

These advantages are achieved without relying on strong assumptions, making ML a valuable tool in clinical practice. Roshan et al. compared the performance of ML and established gold standard scoring tools (POAF score) in predicting POAF during ICU admission after cardiac surgery. The results showed that the ML model outperformed the clinical scoring tools in predicting POAF (21). Parise et al. (22) used four ML algorithms to analyze 394 patients who underwent CABG surgery and compared their performance. They also conducted traditional logistic regression analysis for comparison with the ML models (22). This study identified the GNB model as the optimal predictive tool through a comparative analysis of multiple ML algorithms using a relatively large cohort. The incorporation of genomic data aligned with the individualized stratification principles of precision medicine, providing robust evidence for early-stage, risk-stratified interventions in high-risk populations.

### Limitations

This study has several limitations. First, while genomic analyses have identified genetic variants associated with POAF, the heterogeneity of these variants complicates the predictive utility of single-marker approaches. Second, although our case collection encompassed all patients from our institution, the sample sizes for training, testing, and independent validation sets remain relatively limited. Additionally, the datasets exhibited class imbalance in label variables. To address this, we employed upsampling via the SMOTE algorithm to enhance model robustness and generalizability. Third, as a single-center study focused on East Asian populations, the model’s cross-ethnic and multi-center applicability remains inadequately validated. Key factors contributing to this limitation include: ethnic variations in the distribution of cardiac-specific genetic polymorphisms (e.g., differential expression of POAF-associated risk loci); disparities in surgical techniques (e.g., CABG procedural choices) and postoperative management strategies (e.g., anticoagulation protocols, arrhythmia prevention measures) across healthcare systems. Lastly, although a visualization web-based tool was developed and passed preliminary technical tests, its clinical implementation remains far from realization.

In summary, to address these limitations, our research team has initiated a multi-faceted improvement strategy: expand the testing of disease-associated genetic variants linked to CABG outcomes, leveraging GWAS to identify population-specific (beyond East Asian cohorts) risk loci, thereby refining the genetic underpinnings of the predictive model. Continuously expand our institution’s sample size and collaborate with multiple (tertiary) hospitals nationwide to transition to multi-center research, thereby overcoming single-center limitations and improving clinical applicability of the model. Clinical Implementation of the Web Tool: progress will be advanced in three sequential phases: Technical Validation: Implement rigorous stress testing and other robustness assessments. Clinical Usability Evaluation: Engage cardiac surgeons and nursing staff in standardized tasks (e.g., quantify usability via the System Usability Scale [SUS] questionnaire). Retrospective Clinical Validation: Evaluate the alignment between the tool’s predictive outputs and clinical decision-making using historical CABG case data (e.g., assess consistency via Cohen’s Kappa coefficient).

## Conclusions

This study combines clinical indicators with 21 pharmacogenomic genes to develop a GNB-based predictive model for post-CABG POAF risk. We created an online tool using 11 key indicators to enable personalized risk assessment, aiding preoperative evaluation and treatment planning to reduce AF incidence and complications.

## Data Availability

The raw data supporting the conclusions of this article will be made available by the authors, without undue reservation.
