# Supplementary material for: Machine learning prediction of post-CABG atrial fibrillation using clinical and pharmacogenomic biomarkers
Source: Front Med (Lausanne). 2025 Sep 11;12:1650700. doi: 10.3389/fmed.2025.1650700 (PMC12460236; doi:10.3389/fmed.2025.1650700)
Supplement: Supplementary file 1 [file Table_1.docx]

**Method S1**

A 5 mL venous blood sample was collected from each patient and subjected to multiple PCR reactions. The following steps were performed. PCR Reaction: Add 3 μL of PCR mixture and 2 μL of DNA sample to each well (after mixing, the total volume: 5 μL). SAP Reaction: Add 2 μL of SAP mixture to each well (after mixing, the total volume: 7 μL). EXT Extension Reaction: Add 2 μL of EXT mixture to each well (after mixing, the total volume: 9 μL). Quantification: Add 16 μL of ultrapure water to each well with a sample (quantification total: 25 μL), then quickly centrifuge.

**Method S2**

**Data Cleaning**

Data cleaning was based on the R language ggpairs package, including clinical and variant data Cleaning.

**Clinical Data Cleaning and Feature Dimensionality Reduction**

This included checks for multicollinearity between continuous variables (using the R language car package), imputation of missing values (using the R language mice package), and removal of outliers and duplicates. Additionally, categorical variables were encoded, such as converting categorical variables from characters to 0-1 encoding (using Python’s Pandas package). The Body Mass Index (BMI) was calculated using the formula: BMI = weight / height², where weight is in kilograms and height is in meters. The correlation between the target POAF (classified as sinus rhythm and atrial fibrillation, SR and AF) and feature variables was assessed. In summary, data cleaning ensured the quality and accuracy of the data, and feature dimensionality reduction reduced the complexity of model construction while enhancing clinical applicability.

**Variant Data Cleaning and Feature Dimensionality Reduction**

Given the presence of rare variants in our cohort (potentially introducing genotype distribution bias/imbalanced allele frequencies), we stratified 28 genetic variants into three categories based on genotype frequency imbalances prior to imputation, with distinct analytical approaches implemented for each category:

1. Bias variants: Defined as loci meeting any of the following criteria:

• Monomorphic sites (single genotype across all samples)

• Biallelic loci with minor allele frequency (MAF) < 10

• Triallelic loci where the combined frequency of any two genotypes < 10

These variants were excluded from subsequent analyses.

2. Moderate-bias variants: Defined as either:

• Biallelic loci with MAF ≥ 10

• Triallelic loci containing at least one genotype with frequency < 10

These were dichotomized based on allele frequency thresholds: biallelic variants retained original categorization, while triallelic variants were simplified by collapsing the two least frequent genotypes into a composite category.

3. Non-bias variants: All remaining triallelic loci.

The variant sites were analyzed based on four classical genetic models (Codominance, Dominance, Recessiveness, and Additivity). The four genetic models are represented by alleles A1 and A2, and their definitions is as followed:

The variant sites were analyzed based on four classical genetic models (Codominance, Dominance, Recessiveness, and Additivity). Feature dimensionality reduction was performed using Chi-square tests or Fisher's exact tests. The four genetic models are represented by alleles A1 and A2, defined as follows:

Codominance: In heterozygotes, both alleles exhibit their own phenotypic effects, and these effects are both expressed simultaneously, resulting in an intermediate phenotype. This model is represented by A1A1, A1A2, and A2A2.

Dominance: The phenotypic effect of one allele (the dominant allele) completely or mainly overrides the effect of the other allele (the recessive allele). Heterozygotes and homozygotes for the dominant allele have the same phenotype, represented by A1A1 and A1A2 + A2A2.

Recessiveness: A recessive trait only manifests when an individual carries two identical recessive alleles. Heterozygotes express the dominant trait, and the phenotype is represented by A1A1 + A1A2 and A2A2.

Additivity: It is assumed that the effect of the genotype on the phenotype is linear, meaning that each allele independently contributes to the phenotype and their effects can be summed. The phenotype of heterozygotes falls between that of the two homozygotes, represented by A1A1 + A2A2 and A1A2.

**Model Construction and Evaluation**

On the Python 3.12 platform, using scikit-learn and related packages, eight machine learning models were constructed, and specifically including:

Gaussian Naive Bayes (GNB) is a simple probabilistic classifier based on Bayes' theorem. It assumes that the features are independent of each other and that each feature follows a Gaussian distribution (1).

K-Nearest Neighbors algorithm (KNN) is an instance-based learning method that classifies samples by measuring the distance between different feature values. Specifically, for a given test sample, the KNN algorithm finds the K nearest samples in the training dataset and determines the test sample's class by voting based on the classes of these K nearest neighbors (2).

Logistic Regression (LR) is a widely used linear model for classification tasks, particularly for binary classification problems. It maps the output of linear regression to the range of 0 to 1 using the sigmoid function, which can be interpreted as a probability output (3).

Multi-Layer Perceptron (MLP) is a feedforward artificial neural network that consists of an input layer, one or more hidden layers, and an output layer. Each layer is composed of neurons, and the neurons are connected by weights. MLP is trained using the backpropagation algorithm and can capture nonlinear relationships in the data, making it suitable for complex classification and regression tasks (4).

Random Forest (RF) is a tree-based ensemble learning method that improves the model's predictive ability by constructing multiple decision trees and combining their predictions through voting or averaging. It introduces randomness by sampling not only the data points but also the features used for splitting, which enhances the model's generalization ability and reduces overfitting (5).

Support Vector Machine (SVM) is a binary classification model. Its basic idea is to find a hyperplane that separates samples of different classes while maximizing the distance (i.e., margin) between the two classes and the hyperplane. For non-linearly separable problems, SVM uses a kernel function to map the data into a higher-dimensional space for classification (6).

TabTransformer (TabTF) is a novel deep learning architecture for tabular data. It processes categorical features through transformer layers to capture contextual embeddings, while handling continuous features conventionally. This hybrid approach enables sophisticated feature interactions and outperforms traditional methods. Developed by Google Research, it achieves state-of-the-art results on structured datasets while maintaining interpretability through attention mechanisms. Ideal for classification/regression tasks with mixed data types (7).

XGBoost (XGB) is an ensemble learning algorithm based on Gradient Boosting Decision Trees (GBDT). It improves upon GBDT by introducing regularization terms and second-order derivative information, which enhances the model's performance and generalization ability (8).

**Table S1** Information and clinical significance of 21-gene testing for personalized cardiovascular and cerebrovascular medication

| **Gene** | **RS ID** | **HGVS ID** | **Testing Significance** |
| --- | --- | --- | --- |
| ADRB1 | rs1801253 | c.1165G > C | The ADRB1 gene encodes the β1-adrenergic receptor, which is the target of β-blockers. Polymorphisms in the ADRB1 gene affect drug efficacy, while polymorphisms in the CYP2D6 gene affect drug metabolism. Adjusting drug dosages based on genotypes can enhance efficacy and reduce the occurrence of adverse reactions. |
| CYP2D6 | rs1065852 | c.100C > T |  |
| ACE | rs1799752 | I/D | The ACE gene encodes angiotensin-converting enzyme, which is the target of ACE inhibitors (ACEI). Patients with the ACE gene II and ID genotypes are less responsive to ACEI, requiring an increased dosage or alternative medications; patients with the DD genotype are sensitive to ACEI and can be treated effectively with the standard dosage. |
|  |  |  |  |
|  |  |  |  |
| CYP2C9 | rs1799853, rs1057910 | c.430C > T, c.1075A > C | Polymorphisms in the CYP2C9 and SLCO1B1 genes affect drug metabolism and transport. Adjusting drug dosages based on genotypes can enhance efficacy and reduce the risk of cardiovascular events. Selecting statin medications based on SLCO1B1 genotype can lower the risk of serious adverse reactions. |
|  |  |  |  |
| SLCO1B1 | rs4149056, rs2306283 | c.521T > C, c.388A > G |  |
|  |  |  |  |
| AGTR1 | rs5186 | c.1166A > C | The AGTR1 gene encodes the angiotensin II type 1 receptor, which is the target of angiotensin receptor blockers (ARBs). Polymorphisms in the AGTR1 gene affect drug efficacy. |
|  |  |  |  |
| ADD1 | rs4961 | c.1378G > T | Indicates the risk of myocardial infarction associated with medication use in hypertensive individuals. |
| HLA-B*5801 | rs3134792, rs4713518 | g.31312326T > G, g.32289560A > G | The HLA-B*5801 gene is highly associated with severe skin adverse reactions to allopurinol and has a higher positive rate among Asian populations. Based on test results, severe drug allergic reactions can be avoided. |
| MTHFR | rs1801133 | C677T | Assessing an individual's ability to metabolize folic acid indicates the risk of H-type hypertension and stroke. |
| CYP2C19 | rs4244285, rs4986893, rs12248560 | c.681G > A, c.636G > A, c.-806C > T | Indicates the risk of clopidogrel resistance, guides antiplatelet therapy, and helps avoid insufficient dosage or ineffective medication. |
| GP1BA | rs6065 | c.482C > T | Indicates the risk of aspirin resistance. |
| ITGB3 | rs5918 | c.176T > C |  |
| PTGS1 | rs10306114 | c.-842A > G |  |
| LTC4S | rs730012 | c.-444A > C |  |
| VKORC1 | rs9923231, rs9934438 | c.-1639G > A | Calculate the initial and maintenance doses of warfarin based on genotype to shorten the adjustment period and reduce the risks of thrombosis and bleeding. Guide medication based on the metabolic enzyme genotype for novel anticoagulants. |
| CYP4F2 | rs2108622 | c.1297G > A |  |
| CYP3A5 | rs776746 | c.806-4288C > T |  |
|  |  |  |  |
| ALDH2 | rs671 | c.1510G > A | Assess the metabolism of nitroglycerin to guide its appropriate clinical use and avoid ineffective sublingual administration of nitroglycerin. |
| C11orf65 | rs11212617 | c.175-5285G > T | By analyzing the patient's genotype, medications with a higher response to the drug can be selected to better achieve therapeutic outcomes. |
| KCNJ11 | rs5219 | c.67A > G |  |
| APOE | rs7412, rs429358 | c.526C > T, c.388T > C | Based on the APOE genotype, the efficacy of statins and fibrates can be predicted. |

Note: HGVS ID, Human Genome Variation Society Identifier

**Table S2 The comparison of influencing factors between the SR group and the AF group among POAF**

| **Factors** | **Category** | **POAF** | | **χ^2^/Fisher/Wil** | ***P*-value** | **95 % CI** |
| --- | --- | --- | --- | --- | --- | --- |
|  |  | **SR** | **AF** |  |  |  |
| Sex | M | 334 | 66 | 0.14 | 0.71 |  |
|  | F | 144 | 32 |  |  |  |
| Age |  | 64.50 (58.00, 70.00) | 68.00 (60.00, 72.00) | 26728 | 0.028 * | 3.93e-5, 4.00 |
| BMI |  | 24.47 (22.19, 26.67) | 24.33 (22.27, 27.19) | 23599 | 0.90 | -0.70, 0.80 |
| BloodType | A | 132 | 27 | 2.48 | 0.48 |  |
|  | AB | 64 | 11 |  |  |  |
|  | B | 151 | 26 |  |  |  |
|  | O | 131 | 34 |  |  |  |
| Smoking | N | 281 | 59 | 0.022 | 0.88 |  |
|  | Y | 197 | 39 |  |  |  |
| Drinking | N | 362 | 73 | 0.017 | 0.90 |  |
|  | Y | 116 | 25 |  |  |  |
| Diabetes | N | 298 | 55 | 1.08 | 0.30 |  |
|  | Y | 180 | 43 |  |  |  |
| Hypertension | N | 197 | 39 | 0.022 | 0.88 |  |
|  | Y | 281 | 59 |  |  |  |
| RenalHx | N | 450 | 86 | 4.19 | 0.041 * |  |
|  | Y | 28 | 12 |  |  |  |
| LiverHx | N | 432 | 89 | 3.22e-30 | 1 |  |
|  | Y | 46 | 9 |  |  |  |
| LungHx | N | 387 | 76 | 0.40 | 0.53 |  |
|  | Y | 91 | 22 |  |  |  |
| StrokeHx | N | 344 | 68 | 0.15 | 0.69 |  |
|  | Y | 134 | 30 |  |  |  |
| ValveHx | N | 359 | 53 | 16.63 | 4.54e-05 * |  |
|  | Y | 119 | 45 |  |  |  |
| MIHx | N | 427 | 86 | 0.077 | 0.78 |  |
|  | Y | 51 | 12 |  |  |  |
| APHx | N | 78 | 21 | 1.15 | 0.28 |  |
|  | Y | 400 | 77 |  |  |  |
| CHDHx | N | 31 | 5 | 0.082 | 0.77 |  |
|  | Y | 447 | 93 |  |  |  |
| HFHx | N | 82 | 30 | 8.56 | 0.0034 * |  |
|  | Y | 396 | 68 |  |  |  |
| AFHx | N | 468 | 80 |  | 1.07e-09 * |  |
|  | Y | 10 | 18 |  |  |  |
| ThyroidHx | N | 426 | 85 | 0.26 | 0.61 |  |
|  | Y | 52 | 13 |  |  |  |
| PCIHx | N | 426 | 83 | 1.15 | 0.28 |  |
|  | Y | 52 | 15 |  |  |  |
| AnticoagulantHx | N | 425 | 82 | 1.65 | 0.20 |  |
|  | Y | 53 | 16 |  |  |  |
| LipidMedHx | N | 437 | 86 | 0.91 | 0.34 |  |
|  | Y | 41 | 12 |  |  |  |
| AntiDiabeticHx | N | 376 | 72 | 0.99 | 0.32 |  |
|  | Y | 102 | 26 |  |  |  |
| BPMedHx | N | 316 | 57 | 1.92 | 0.17 |  |
|  | Y | 162 | 41 |  |  |  |
| CABGVx | < 3 | 103 | 33 | 5.97 | 0.015 * |  |
|  | ≥ 3 | 375 | 65 |  |  |  |
| CABGTime |  | 230.00 (205.00, 276.50) | 250.00 (217.80, 297.80) | 28395 | 0.00092 * | 9.00, 35.00 |

Note *: *P* < 0.05; AF: atrial fibrillation; SR: sinus rhythm

**Table S3 The comparison of variant factors between the SR group and the AF group among POAF for non-bias variants**

| **Factors** | **Codominant model** | | | | **Recessive model** | | | | **Dominant model** | | | | **Additive model** | | | | **HWE** | |
| --- | --- | --- | --- | --- | --- | --- | --- | --- | --- | --- | --- | --- | --- | --- | --- | --- | --- | --- |
|  | **Geno** | **SR** | **AF** | ***P*-value** | **Geno** | **SR** | **AF** | ***P*-value** | **Geno** | **SR** | **AF** | ***P*-value** | **Geno** | **SR** | **AF** | ***P*-value** | **χ^2^** | ***P*-value** |
| rs1065852/  CYP2D6  CYP2D6 | CC | 136 | 28 | 0.39 | CC + CT | 361 | 68 | 0.25 | CC | 136 | 28 | 1.00 | CC + TT | 253 | 58 | 0.31 | 3.60 | 0.06 |
|  | CT | 225 | 40 |  | TT | 117 | 30 |  | CT + TT | 342 | 70 |  | CT | 225 | 40 |  |  |  |
|  | TT | 117 | 30 |  |  |  |  |  |  |  |  |  |  |  |  |  |  |  |
| rs1799752/  ACE | DD | 69 | 15 | 0.91 | DD + DI | 285 | 57 | 0.88 | DD | 69 | 15 | 0.95 | DD + II | 262 | 56 | 0.76 | 0.88 | 0.37 |
|  | DI | 216 | 42 |  | II | 193 | 41 |  | DI + II | 409 | 83 |  | DI | 216 | 42 |  |  |  |
|  | II | 193 | 41 |  |  |  |  |  |  |  |  |  |  |  |  |  |  |  |
| rs5219/  KCNJ11 | AA | 78 | 24 | 0.15 | AA + GA | 311 | 68 | 0.48 | AA | 78 | 24 | 0.074 * | AA + GG | 245 | 54 | 0.56 | 0.07 | 0.80 |
|  | GA | 233 | 44 |  | GG | 167 | 30 |  | GA + GG | 400 | 74 |  | GA | 233 | 44 |  |  |  |
|  | GG | 167 | 30 |  |  |  |  |  |  |  |  |  |  |  |  |  |  |  |
| rs730012/  LTC4S | AA | 348 | 74 | 0.13 | AA + CA | 465 | 92 | 0.11 | AA | 348 | 74 | 0.67 | AA + CC | 361 | 80 | 0.24 | 3.82 | 0.06 |
|  | CA | 117 | 18 |  | CC | 13 | 6 |  | CA + CC | 130 | 24 |  | CA | 117 | 18 |  |  |  |
|  | CC | 13 | 6 |  |  |  |  |  |  |  |  |  |  |  |  |  |  |  |
| rs2108622/  CYP4F2 | AA | 43 | 6 | 0.31 | AA + AG | 235 | 53 | 0.44 | AA | 43 | 6 | 0.47 | AA + GG | 286 | 51 | 0.19 | 0.01 | 0.93 |
|  | AG | 192 | 47 |  | GG | 243 | 45 |  | AG + GG | 435 | 92 |  | AG | 192 | 47 |  |  |  |
|  | GG | 243 | 45 |  |  |  |  |  |  |  |  |  |  |  |  |  |  |  |
| rs776746/  CYP3A5 | CC | 238 | 46 | 0.11 | CC + TC | 445 | 85 | 0.056 * | CC | 238 | 46 | 0.69 | CC + TT | 271 | 59 | 0.60 | 0.52 | 0.49 |
|  | TC | 207 | 39 |  | TT | 33 | 13 |  | TC + TT | 240 | 52 |  | TC | 207 | 39 |  |  |  |
|  | TT | 33 | 13 |  |  |  |  |  |  |  |  |  |  |  |  |  |  |  |
| rs4713518/  HLA-B*5801 | AA | 130 | 16 | 0.079 * | AA + GA | 364 | 71 | 0.52 | AA | 130 | 16 | 0.034 * | AA +GG | 244 | 43 | 0.24 | 0.01 | 0.94 |
|  | GA | 234 | 55 |  | GG | 114 | 27 |  | GA + GG | 348 | 82 |  | GA | 234 | 55 |  |  |  |
|  | GG | 114 | 27 |  |  |  |  |  |  |  |  |  |  |  |  |  |  |  |
| rs2306283/  SLCO1B1 | AA | 33 | 7 | 0.98 | AA + GA | 224 | 45 | 0.95 | AA | 33 | 7 | 1.00 | AA + GG | 287 | 60 | 0.92 | 0.09 | 0.75 |
|  | GA | 191 | 38 |  | GG | 254 | 53 |  | GA + GG | 445 | 91 |  | GA | 191 | 38 |  |  |  |
|  | GG | 254 | 53 |  |  |  |  |  |  |  |  |  |  |  |  |  |  |  |
| rs11212617/  C11orf65 | GG | 177 | 37 | 0.99 | GG + GT | 395 | 81 | 1.00 | GG | 177 | 37 | 0.98 | GG + TT | 260 | 54 | 0.99 | 1.63 | 0.22 |
|  | GT | 218 | 44 |  | TT | 83 | 17 |  | GT + TT | 301 | 61 |  | GT | 281 | 44 |  |  |  |
|  | TT | 83 | 17 |  |  |  |  |  |  |  |  |  |  |  |  |  |  |  |
| rs4244285/  CYP2C19 | AA | 37 | 9 | 0.86 | AA + GA | 242 | 49 | 1.00 | AA | 37 | 9 | 0.78 | AA + GG | 273 | 58 | 0.79 | 0.44 | 0.49 |
|  | GA | 205 | 40 |  | GG | 236 | 49 |  | GA + GG | 441 | 89 |  | GA | 205 | 40 |  |  |  |
|  | GG | 236 | 49 |  |  |  |  |  |  |  |  |  |  |  |  |  |  |  |
| rs4961/  ADD1 | GG | 105 | 13 | 0.11 | GG + GT | 347 | 72 | 0.96 | GG | 105 | 13 | 0.071 * | GG + TT | 236 | 39 | 0.11 | 1.44 | 0.21 |
|  | GT | 242 | 59 |  | TT | 131 | 26 |  | GT + TT | 373 | 85 |  | GT | 242 | 59 |  |  |  |
|  | TT | 131 | 26 |  |  |  |  |  |  |  |  |  |  |  |  |  |  |  |
| rs1801253/  ADRB1 | CC | 260 | 52 | 0.97 | CC + GC | 449 | 92 | 1.00 | CC | 260 | 52 | 0.90 | CC + GG | 289 | 58 | 0.90 | 0.68 | 0.39 |
|  | GC | 189 | 40 |  | GG | 29 | 6 |  | GC + GG | 218 | 46 |  | GC | 189 | 40 |  |  |  |
|  | GG | 29 | 6 |  |  |  |  |  |  |  |  |  |  |  |  |  |  |  |
| rs1801133/  MTHFR | CC | 68 | 16 | 0.74 | CC + CT | 277 | 55 | 0.82 | CC | 68 | 16 | 0.70 | CC + TT | 269 | 59 | 0.55 | 2.58 | 0.13 |
|  | CT | 209 | 39 |  | TT | 201 | 43 |  | CT + TT | 410 | 82 |  | CT | 209 | 39 |  |  |  |
|  | TT | 201 | 43 |  |  |  |  |  |  |  |  |  |  |  |  |  |  |  |

Note *: *P* < 0.10; AF: atrial fibrillation; SR: sinus rhythm; HWE: Hardy-Weinberg Equilibrium; Geno: Genotype

**Table S4** The comparison of variant factors between the SR group and the AF group among POAF for moderate-bias variants

| **Factors** | **Geno** | **SR** | **AF** | ***P*-value** |
| --- | --- | --- | --- | --- |
| rs3134792 | GG + GT | 53 | 8 | 0.50 |
|  | TT | 425 | 90 |  |
| rs4149056 | CC + CT | 96 | 20 | 1.00 |
|  | TT | 382 | 78 |  |
| rs7412 | CC | 432 | 86 | 0.55 |
|  | TT + CT | 46 | 12 |  |
| rs12248560 | CC | 464 | 94 | 0.53 |
|  | CT | 14 | 4 |  |
| rs4986893 | AA + GA | 58 | 10 | 0.71 |
|  | GG | 420 | 88 |  |
| rs9934438 | TC | 91 | 20 | 0.86 |
|  | TT | 387 | 78 |  |
| rs9923231 | AA | 388 | 79 | 1.00 |
|  | GG + GA | 90 | 19 |  |
| rs1057910 | AA | 441 | 90 | 1.00 |
|  | CC + CA | 37 | 8 |  |
| rs671 | GA | 132 | 27 | 1.00 |
|  | GG | 346 | 71 |  |
| rs429358 | CC + CT | 70 | 16 | 0.79 |
|  | TT | 408 | 82 |  |
| rs5186 | AA | 403 | 80 | 0.61 |
|  | CC + CA | 75 | 18 |  |
| rs6065 | CC | 413 | 88 | 0.46 |
|  | TT + CT | 65 | 10 |  |

**Table S5** Genotype frequency table of mutation loci prior to interpolation processing

| Variants | | |  |  |  |
| --- | --- | --- | --- | --- | --- |
| ===== rs1065852 ===== | | |  |  |  |
| CC CT TT | |  |  |  |  |
| 156 249 141 | |  |  |  |  |
|  |  |  |  |  |  |
| ===== rs3134792 ===== | | |  |  |  |
| GG GT TT | |  |  |  |  |
| 5 52 492 | |  |  |  |  |
|  |  |  |  |  |  |
| ===== rs1799752 ===== | | |  |  |  |
| DD DI II |  |  |  |  |  |
| 81 246 222 | |  |  |  |  |
|  |  |  |  |  |  |
| ===== rs5219 ===== | | |  |  |  |
| AA GA GG | |  |  |  |  |
| 94 268 187 | |  |  |  |  |
|  |  |  |  |  |  |
| ===== rs730012 ===== | | |  |  |  |
| AA CA CC | |  |  |  |  |
| 409 123 17 | |  |  |  |  |
|  |  |  |  |  |  |
| ===== rs4149056 ===== | | |  |  |  |
| CC CT TT | |  |  |  |  |
| 4 103 442 | |  |  |  |  |
|  |  |  |  |  |  |
| ===== rs2108622 ===== | | |  |  |  |
| AA AG GG | |  |  |  |  |
| 44 227 278 | |  |  |  |  |
|  |  |  |  |  |  |
| ===== rs7412 ===== | | |  |  |  |
| CC CT TT | |  |  |  |  |
| 498 50 1 | |  |  |  |  |
|  |  |  |  |  |  |
| ===== rs776746 ===== | | |  |  |  |
| CC TC TT | |  |  |  |  |
| 271 233 45 | |  |  |  |  |
|  |  |  |  |  |  |
| ===== rs4713518 ===== | | | |  |  |
| AA GA GG | |  |  |  |  |
| 138 278 133 | |  |  |  |  |
|  |  |  |  |  |  |
| ===== rs2306283 ===== | | |  |  |  |
| AA GA GG | |  |  |  |  |
| 37 218 294 | |  |  |  |  |
|  |  |  |  |  |  |
| ===== rs12248560 ===== | | |  |  |  |
| CC CT |  |  |  |  |  |
| 538 11 |  |  |  |  |  |
|  |  |  |  |  |  |
| ===== rs11212617 ===== | | |  |  |  |
| GG GT TT | |  |  |  |  |
| 204 254 91 | |  |  |  |  |
|  |  |  |  |  |  |
| ===== rs4986893 ===== | | |  |  |  |
| AA GA GG | |  |  |  |  |
| 1 64 484 | |  |  |  |  |
|  |  |  |  |  |  |
| ===== rs9934438 ===== | | | |  |  |
| TC TT |  |  |  |  |  |
| 85 464 |  |  |  |  |  |
|  |  |  |  |  |  |
| ===== rs4244285 ===== | | |  |  |  |
| AA GA GG | |  |  |  |  |
| 41 236 272 | |  |  |  |  |
|  |  |  |  |  |  |
| ===== rs9923231 ===== | | |  |  |  |
| AA GA GG | |  |  |  |  |
| 466 81 2 | |  |  |  |  |
|  |  |  |  |  |  |
| =====rs4961 ===== | | |  |  |  |
| GG GT TT | |  |  |  |  |
| 112 285 152 | |  |  |  |  |
|  |  |  |  |  |  |
| ===== rs1057910 ===== | | | |  |  |
| AA CA CC | |  |  |  |  |
| 507 38 4 | |  |  |  |  |
|  |  |  |  |  |  |
| ===== rs671 ===== | | |  |  |  |
| GA GG |  |  |  |  |  |
| 154 395 |  |  |  |  |  |
|  |  |  |  |  |  |
| ===== rs429358 ===== | | |  |  |  |
| CC CT TT | |  |  |  |  |
| 1 83 465 | |  |  |  |  |
|  |  |  |  |  |  |
| ===== rs1801253 ===== | | |  |  |  |
| CC GC GG | |  |  |  |  |
| 298 217 34 | |  |  |  |  |
|  |  |  |  |  |  |
| ===== rs5186 ===== | | |  |  |  |
| AA CA CC | |  |  |  |  |
| 467 79 3 | |  |  |  |  |
|  |  |  |  |  |  |
| ===== rs6065 ===== | | |  |  |  |
| CC CT TT | |  |  |  |  |
| 478 69 2 | |  |  |  |  |
|  |  |  |  |  |  |
| ===== rs1801133 ===== | | |  |  |  |
| CC CT TT | |  |  |  |  |
| 81 237 231 | |  |  |  |  |
|  |  |  |  |  |  |
| ===== rs5918 ===== | | |  |  |  |
| CC CT TT | |  |  |  |  |
| 1 8 540 | |  |  |  |  |
|  |  |  |  |  |  |
| ===== rs10306114 ===== | | |  |  |  |
| A AG |  |  |  |  |  |
| 548 1 |  |  |  |  |  |
|  |  |  |  |  |  |
| ===== rs1799853 ===== | | |  |  |  |
| C |  |  |  |  |  |
| 549 |  |  |  |  |  |

**Table S6** The hyperparameter sets for eight training models

| **ParameterGid** | **AlgorithmModel** | | | | | | | |
| --- | --- | --- | --- | --- | --- | --- | --- | --- |
|  | **GNB** | **KNN** | **LR** | **MLP** | **RF** | **SVM** | **TabTF** | **XGB** |
| **n_estimators** |  |  |  |  | 50, 150, 200, 300 |  |  | 200, 300, 500 |
| **max_depth** |  |  |  |  | 3, 4, 5, 7 |  |  | 3, 5, 7, 9 |
| **min_samples_split** |  |  |  |  | 2, 3, 5, 7 |  |  |  |
| **min_samples_leaf** |  |  |  |  | 1, 2, 3, 4, 5 |  |  |  |
| **learning_rate** |  |  |  |  |  |  | [0.001, 0.0001] | 0.1, 0.3, 0.5 |
| **colsample_bytree** |  |  |  |  |  |  |  | 0.4, 0.6, 1 |
| **min_child_weight** |  |  |  |  |  |  |  | 1, 2, 4 |
| **subsample** |  |  |  |  |  |  |  | 0.5, 0.7, 1.0 |
| **var_smoothing** | np.logspace(-9, 0, 30) |  |  |  |  |  |  |  |
| **n_neighbors** |  | 3, 5, 7, 11 |  |  |  |  |  |  |
| **weights** |  | 'uniform', 'distance' |  |  |  |  |  |  |
| **metric** |  | 'euclidean', 'manhattan' |  |  |  |  |  |  |
| **C** |  |  | 0.001, 0.01, 0.1, 1, 10, 100 |  |  | 0.1, 1, 10 |  |  |
| **solver** |  |  | 'liblinear', 'saga' |  |  |  |  |  |
| **penalty** |  |  | 'l1', 'l2' |  |  |  |  |  |
| **hidden_layer_sizes** |  |  |  | (16, 32, 1), (32, 16, 1),(10, 30, 1) |  |  |  |  |
| **activation** |  |  |  | 'relu', 'tanh', 'logistic' |  |  |  |  |
| **solver** |  |  |  | 'lbfgs', 'sgd', 'adam' |  |  |  |  |
| **learning_rate_init** |  |  |  | 0.001, 0.01, 0.1 |  |  |  |  |
| **batch_size** |  |  |  | 'auto', 10, 20, 50 |  |  |  |  |
| **max_iter** |  |  |  | 300, 500 |  |  |  |  |
| **kernel** |  |  |  |  |  | 'linear', 'rbf' |  |  |
| **gamma** |  |  |  |  |  | 0.001, 0.01, 0.1 |  |  |
| **embedding_dim** |  |  |  |  |  |  | [8, 16] |  |
| **num_heads** |  |  |  |  |  |  | [2, 4] |  |
| **num_layers** |  |  |  |  |  |  | [1, 2] |  |
| **hidden_dim** |  |  |  |  |  |  | [64, 128] |  |
| **batch_size** |  |  |  |  |  |  | [16, 32] |  |
| **dropout** |  |  |  |  |  |  | [0.1, 0.2] |  |
| **weight_decay** |  |  |  |  |  |  | [1e-4, 1e-5] |  |
| **epochs** |  |  |  |  |  |  | 50 |  |

Note: np.logspace: logspace function in python numpy package; GNB: Gaussian Naive Bayes; KNN: K-Nearest Neighbors algorithm; LR: Logistic Regression; MLP: Multi-Layer Perceptron; RF: Random Forest; SVM: Support Vector Machine; TabTF: TabTransformer; XGB: XGBoost.

**Table S7** The optimal parameters of eight training models

| **AlgorithmModel** | **OptimalParameter** |
| --- | --- |
| **GNB** | 'var_smoothing': 5.30e-05 |
| **KNN** | 'metric': 'manhattan', 'n_neighbors': 11, 'weights': 'distance' |
| **LR** | 'C': 0.001, 'penalty': 'l2', 'solver': 'liblinear' |
| **MLP** | 'activation': 'relu', 'batch_size': 20, 'hidden_layer_sizes': (32, 16, 1), 'learning_rate_init': 0.01, 'max_iter': 300, 'random_state': 42, 'solver': 'sgd' |
| **RF** | 'max_depth': 7, 'min_samples_leaf': 1, 'min_samples_split': 7, 'n_estimators': 150 |
| **SVM** | 'C': 1, 'gamma': 0.1, 'kernel': 'rbf' |
| **TabTF** | 'weight_decay': 1e-05, 'num_layers': 1, 'num_heads': 4, 'lr': 0.001, 'hidden_dim': 128, 'best_epochs': 12, 'embedding_dim': 16, 'dropout': 0.1, 'batch_size': 16 |
| **XGB** | 'colsample_bytree': 1, 'learning_rate': 0.1, 'max_depth': 9, 'min_child_weight': 2, 'n_estimators': 500, 'subsample': 0.7 |

**Table S8** Evaluation parameters of eight models on test set and independent validation set

| **Model** | **Test set** | | **Independent validation set** | | **Mean** | | |
| --- | --- | --- | --- | --- | --- | --- | --- |
|  | **Accuracy** | **AUC** | **Accuracy** | **AUC** | **Accuracy** | | **AUC** |
| **GNB** | 0.81 | 0.81 (0.70, 0.91) | 0.79 | 0.76 (0.62, 0.89) | 0.80 | 0.79 | |
| **KNN** | 0.67 | 0.71 (0.59, 0.82) |  |  |  |  | |
| **LR** | 0.64 | 0.78 (0.66, 0.88) |  |  |  |  | |
| **MLP** | 0.65 | 0.67 (0.58, 0.81) |  |  |  |  | |
| **RF** | 0.74 | 0.74 (0.63, 0.83) |  |  |  |  | |
| **SVM** | 0.68 | 0.71 (0.59, 0.81) |  |  |  |  | |
| **TabTF** | 0.76 | 0.79 (0.69, 0.88) |  |  |  |  | |
| **XGB** | 0.71 | 0.68 (0.54, 0.77) |  |  |  |  | |

**References**

1. Ontivero-Ortega M, Lage-Castellanos A, Valente G, Goebel R, Valdes-Sosa M. Fast Gaussian Naive Bayes for searchlight classification analysis. Neuroimage. 2017;163:471-9.

2. Sarkar M, Leong TY. Application of K-nearest neighbors algorithm on breast cancer diagnosis problem. Proc AMIA Symp. 2000:759-63.

3. Boni YT, Cammarota RC, Liao K, Sigman MS, Davies HML. Leveraging Regio- and Stereoselective C(sp(3))-H Functionalization of Silyl Ethers to Train a Logistic Regression Classification Model for Predicting Site-Selectivity Bias. J Am Chem Soc. 2022;144(34):15549-61.

4. Lorencin I, Andelic N, Spanjol J, Car Z. Using multi-layer perceptron with Laplacian edge detector for bladder cancer diagnosis. Artif Intell Med. 2020;102:101746.

5. Feng H, Wang F, Li N, Xu Q, Zheng G, Sun X, et al. A Random Forest Model for Peptide Classification Based on Virtual Docking Data. Int J Mol Sci. 2023;24(14).

6. Moosaei H, Ganaie MA, Hladik M, Tanveer M. Inverse free reduced universum twin support vector machine for imbalanced data classification. Neural Netw. 2023;157:125-35.

7. Khan MA. A Comparative Study on Imputation Techniques: Introducing a Transformer Model for Robust and Efficient Handling of Missing EEG Amplitude Data. Bioengineering (Basel). 2024;11(8).

8. Shi J, Chen L, Yuan X, Yang J, Xu Y, Shen L, et al. A potential XGBoost Diagnostic Score for Staphylococcus aureus bloodstream infection. Front Immunol. 2025;16:1574003.

**Code S1**

def cross_validate(X, y, params):

SEED = params.get('seed', 377)

torch.manual_seed(SEED)

np.random.seed(SEED)

if torch.cuda.is_available():

torch.cuda.manual_seed_all(SEED)

torch.backends.cudnn.deterministic = True

torch.backends.cudnn.benchmark = False

skf = StratifiedKFold(n_splits=10, shuffle=True, random_state=SEED)

fold_results = []

early_stop_epochs = []

num_categories = [X[col].nunique() for col in cat_features]

for fold, (train_idx, val_idx) in enumerate(skf.split(X, y)):

print(f"\nFold {fold+1}/10")

X_train_fold, X_val_fold = X.iloc[train_idx], X.iloc[val_idx]

y_train_fold, y_val_fold = y.iloc[train_idx], y.iloc[val_idx]

train_dataset = HeartDataset(X_train_fold, y_train_fold)

val_dataset = HeartDataset(X_val_fold, y_val_fold)

fold_seed = SEED + fold

g = torch.Generator()

g.manual_seed(fold_seed)

train_loader = DataLoader(

train_dataset,

batch_size=params['batch_size'],

shuffle=True,

generator=g

)

val_loader = DataLoader(

val_dataset,

batch_size=params['batch_size'],

shuffle=False

)

model = TabTransformer(

num_categories=num_categories,

embedding_dim=params['embedding_dim'],

num_heads=params['num_heads'],

num_layers=params['num_layers'],

hidden_dim=params['hidden_dim'],

dropout=params['dropout']

).to(device)

optimizer = optim.Adam(

model.parameters(),

lr=params['lr'],

weight_decay=params['weight_decay']

)

criterion = nn.BCELoss()

best_val_acc = 0

best_epoch = 0

no_improve_count = 0

patience = params.get('patience', 5)

stopped_epoch = params['epochs']

for epoch in range(params['epochs']):

epoch_seed = fold_seed + epoch

torch.manual_seed(epoch_seed)

if torch.cuda.is_available():

torch.cuda.manual_seed(epoch_seed)

train_loss = train_model(model, train_loader, optimizer, criterion)

val_acc, val_auc = evaluate_model(model, val_loader)

print(f"Epoch {epoch+1}/{params['epochs']} | "

f"Train Loss: {train_loss:.4f} | "

f"Val Acc: {val_acc:.4f} | Val AUC: {val_auc:.4f}")

if val_acc > best_val_acc:

best_val_acc = val_acc

best_epoch = epoch

best_model_state = model.state_dict().copy()

no_improve_count = 0

print(f"↑ Improvement! Saving model (Acc: {val_acc:.4f})")

else:

no_improve_count += 1

print(f"No improvement ({no_improve_count}/{patience})")

if no_improve_count >= patience:

stopped_epoch = epoch + 1

print(f"Early stopping at epoch {epoch+1}. "

f"No improvement for {patience} consecutive epochs.")

print(f"Best validation accuracy: {best_val_acc:.4f} at epoch {best_epoch+1}")

break

early_stop_epochs.append(stopped_epoch)

print(f"Fold {fold+1} stopped at epoch {stopped_epoch}")

model.load_state_dict(best_model_state)

final_val_acc, final_val_auc = evaluate_model(model, val_loader)

fold_results.append(final_val_acc)

print(f"Fold {fold+1} Best Val Acc: {final_val_acc:.4f}, Best Val AUC: {final_val_auc:.4f}")

mean_val_acc = np.mean(fold_results)

mean_early_stop_epoch = np.mean(early_stop_epochs)

print(f"\nCross-validation completed. "

f"Mean Val Acc: {mean_val_acc:.4f}, "

f"Mean Early Stop Epoch: {mean_early_stop_epoch:.1f}")

return mean_val_acc, mean_early_stop_epoch

param_grid = {

'embedding_dim': [8, 16],

'num_heads': [2, 4],

'num_layers': [1, 2],

'hidden_dim': [64, 128],

'lr': [0.001, 0.0001],

'batch_size': [16, 32],

'dropout': [0.1, 0.2],

'weight_decay': [1e-4, 1e-5],

'epochs': [50],

'seed': [377]

}

n_iter = 30

param_sampler = ParameterSampler(param_grid, n_iter=n_iter, random_state=77)

params_list = list(param_sampler)

best_score = 0

best_params = None

best_epoch = 0

results = []

print(f"Starting random grid search with {n_iter} combinations...")

for i, params in enumerate(params_list):

print(f"\nTesting combination {i+1}/{n_iter}:")

print("Parameters:", {k: v for k, v in params.items() if k != 'seed'})

score, avg_early_stop = cross_validate(X_train, y_train, params)

results.append({

'params': params,

'score': score,

'avg_early_stop': avg_early_stop

})

print(f"Average Validation Accuracy: {score:.4f}, Average Early Stop Epoch: {avg_early_stop:.1f}")

if score > best_score:

best_score = score

best_params = params

best_epoch = round(avg_early_stop)

print(f"★ New best! Score: {score:.4f}")

print("\nRandom grid search completed.")

print(f"Total combinations tested: {len(results)}")

print(f"\nBest Parameters: {best_params}")

print(f"Best Validation Accuracy: {best_score:.4f}")

print(f"Recommended Training Epochs: {int(best_epoch * 1.2)}")
